# Microbial activity in the marine deep biosphere: progress and prospects

**DOI:** 10.3389/fmicb.2013.00189

**Published:** 2013-07-11

**Authors:** Beth N. Orcutt, Douglas E. LaRowe, Jennifer F. Biddle, Frederick S. Colwell, Brian T. Glazer, Brandi Kiel Reese, John B. Kirkpatrick, Laura L. Lapham, Heath J. Mills, Jason B. Sylvan, Scott D. Wankel, C. Geoff Wheat

**Affiliations:** ^1^Bigelow Laboratory for Ocean SciencesEast Boothbay, ME, USA; ^2^Department of Earth Sciences, University of Southern CaliforniaLos Angeles, CA, USA; ^3^College of Earth, Ocean and Environment, University of DelawareLewes, DE, USA; ^4^College of Earth, Ocean and Atmospheric Sciences, Oregon State UniversityOR, USA; ^5^Department of Oceanography, University of Hawai'i and ManoaHonolulu, HI, USA; ^6^Marine and Environmental Biology, University of Southern CaliforniaLos Angeles, CA, USA; ^7^Graduate School of Oceanography, University of Rhode IslandNarragansett, RI, USA; ^8^Chesapeake Biological Laboratory, University of Maryland Center for Environmental SciencesSolomons, MD, USA; ^9^Department of Biology, University of Houston Clear LakeTX, USA; ^10^Marine Chemistry and Geochemistry, Woods Hole Oceanographic InstitutionWoods Hole, MA, USA; ^11^Global Undersea Research Unit, University of Alaska FairbanksMoss Landing, CA, USA

**Keywords:** deep biosphere, IODP, biogeochemistry, sediment, oceanic crust, C-DEBI, subsurface microbiology

## Abstract

The vast marine deep biosphere consists of microbial habitats within sediment, pore waters, upper basaltic crust and the fluids that circulate throughout it. A wide range of temperature, pressure, pH, and electron donor and acceptor conditions exists—all of which can combine to affect carbon and nutrient cycling and result in gradients on spatial scales ranging from millimeters to kilometers. Diverse and mostly uncharacterized microorganisms live in these habitats, and potentially play a role in mediating global scale biogeochemical processes. Quantifying the rates at which microbial activity in the subsurface occurs is a challenging endeavor, yet developing an understanding of these rates is essential to determine the impact of subsurface life on Earth's global biogeochemical cycles, and for understanding how microorganisms in these “extreme” environments survive (or even thrive). Here, we synthesize recent advances and discoveries pertaining to microbial activity in the marine deep subsurface, and we highlight topics about which there is still little understanding and suggest potential paths forward to address them. This publication is the result of a workshop held in August 2012 by the NSF-funded Center for Dark Energy Biosphere Investigations (C-DEBI) “theme team” on microbial activity (www.darkenergybiosphere.org).

## What is the marine deep biosphere and why is it important?

In 1992, Thomas Gold presented the thought: “If there exists this deep, hot biosphere, it will become a central item in the discussion of many, or indeed most, branches of the Earth sciences. How much of the biological imprint of material in the sediments is due to surface life and how much to life at depth?” (Gold, 1992). Since the early days of deep biosphere research, the challenge of understanding the scope, relevance and activity of subsurface life has remained a somewhat daunting task, considering that the deep biosphere inhabits the majority of our planet yet is relatively difficult to access. The marine deep biosphere is often defined as life existing deeper than one meter below seafloor (Jørgensen and Boetius, 2007), spanning from continental margins to abyssal plains. Environments in the dark reaches of ocean depths, such as hydrothermal vent systems and newly formed oceanic crust (Orcutt et al., 2011a; Biddle et al., 2012) are also part of the marine deep biosphere, though more often viewed as “windows” to subsurface ecosystems (Deming and Baross, 1993; Huber et al., 2006; Santelli et al., 2008). While recent estimates for the number of microorganisms living in the sedimentary deep biosphere have considerably decreased (Kallmeyer et al., 2012), the number of microbes in the crustal environment is still largely unconstrained (Edwards et al., 2012a), and the vastness of this ecosystem means that it is a major reservoir for harboring microbial life on this planet. However, as Gold postulated in 1992, intriguing questions remain as to the activity that exists in the deep biosphere, both hot and cold.

**Figure 1:**
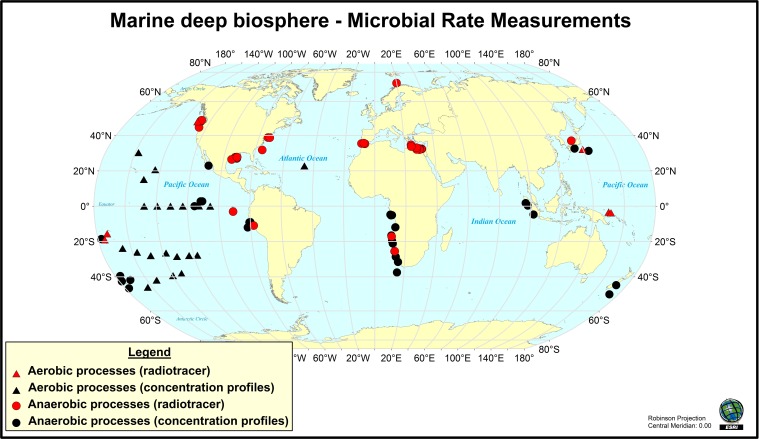
**Map of the locations where rates of microbial activity in deep sediment have been measured (through radio-isotope tracer techniques) or inferred from modeling of vertical geochemical parameters during drilling program expeditions**. This map does not include hydrocarbon seep environments. Map created using ArcGIS 9. References used are (Oremland et al., 1982; Tarafa et al., 1986; Whelan et al., 1986; Cragg et al., 1990, 1992; de Angelis et al., 1993; Cragg et al., 1995; Lein et al., 1997; Fossing et al., 2000; Hoehler et al., 2000; Tsunogai et al., 2000; D'Hondt et al., 2002; 2004, 2009; Böttcher et al., 2004; Joye et al., 2004, 2009; Lam et al., 2004, 2008; Orcutt et al., 2005; Parkes et al., 2005; Niemann et al., 2006a; Sivan et al., 2007; Wang et al., 2008; Nunoura et al., 2009; Omoregie et al., 2009; Schippers et al., 2010; Wankel et al., 2010, 2011; Yoshioka et al., 2010; Lomstein et al., 2012; Nickel et al., 2012; Røy et al., 2012; Ziebis et al., 2012; Maignien et al., 2013).

**Figure 2:**
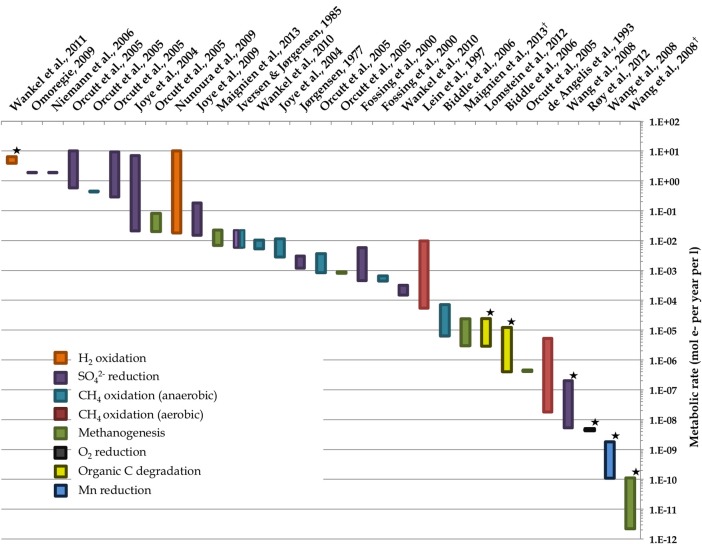
**Representative ranges of microbial activity in the marine deep biosphere based on literature values of measured and modeled volumetric rates**. Starred rate measurements derive from measurements of *in situ* conditions; all others derive from *ex situ* incubation experiments. Note that the value from Wankel et al. (2011) assumes a depth of 10 cm. Environments include some deep sediment locales as well as “windows” to the deep biosphere such as hydrothermal vents. Rates of different metabolisms are normalized to moles of electrons per unit time per unit volume. Dagger denotes references where the ranges of bar in graph reflect the 8-fold difference in moles of electrons per methane molecule for methanogenesis depending on the substrate—8 mol e- per 1 mol CO_2_ vs. 1 mol e- per 1 mol acetate. References used are (de Angelis et al., 1993; Iversen and Jørgensen 1985, Jørgensen, 1977; Lein et al., 1997; Fossing et al., 2000; Joye et al., 2004, 2009; Orcutt et al., Biddle et al., 2006b; Niemann et al., 2006b; Wang et al., 2008; Nunoura et al., 2009; Omoregie et al., 2009; Wankel et al., 2010, 2011; Lomstein et al., 2012; Røy et al., 2012; Maignien et al., 2013).

Importantly, the marine deep biosphere is alive: it is not just a reservoir for buried, non-functioning microbial cells. Evidence of the activity of microorganisms in the marine deep biosphere comes from numerous angles, such as geochemical profiles (Oremland et al., 1982; D'Hondt et al., 2002; Røy et al., 2012), enumeration of cells (Cragg et al., 1990, 1992; Cragg and Kemp, 1995), extraction of RNA from deep sediment [thought to only derive from live cells; (Mills et al., 2012a; Orsi et al., 2013a,b)], extraction of intact polar lipids from deep sediment [again, thought to only derive from live cells; (Lipp et al., 2008)], ability to enrich indigenous microorganisms with stable isotope labeling (Morono et al., 2011), growth of viable cultures and enrichments (Cragg et al., 1990; D'Hondt et al., 2004; Batzke et al., 2007; Smith et al., 2011), and measurements of substrate turnover (Figures 1, 2). However, these data have also come with controversy, such as cross-laboratory comparisons yielding different results (Biddle et al., 2006a; Schippers and Neretin, 2006; Lipp et al., 2008), extraction biases and efficiency issues (Mills et al., 2012a,b), persistence of biomolecules like intact polar lipids in the environment (Schouten et al., 2010, 2013; Logemann et al., 2011; Xie et al., 2013), thermogenic activity influencing deep metabolisms (Parkes et al., 2011), and calculated rates of per cell activity that are orders of magnitude lower than anything known through cultivation (Jørgensen and Boetius, 2007; D'Hondt et al., 2009; Røy et al., 2012; Hoehler and Jørgensen, 2013). These controversial data are challenging our notions of what it means to “be alive” and “active” vs. dormant or “dead” (Jørgensen, 2011, 2012) [some have even said “zombie,” (Colwell and D'Hondt, 2013)].

The realization that microbes exist in the deep biosphere, and are active to some degree, brings to question the importance of understanding this activity. It is generally assumed that metabolic rates in the deep biosphere are low, yet potentially capable of influencing important global biogeochemical cycles (elements such as C, H, O, N, Fe, Mn, S). For example, sedimentary microbial processes account for oxidation of 95% of the methane that exists in marine sediment, reducing the amount of methane flux to the water column (Reeburgh et al., 1993; Reeburgh, 1996). Yet, microbial activity is low enough to allow geochemical and paleooceanography proxies to persist (Meyers, 1997; Zachos et al., 2001), although, areas of microbial activity can be high enough to destroy these proxies (Shah et al., 2008). Understanding how microbial activity in the subsurface can influence well-established, or potentially new, proxies for paleo-oceanography will help to better understand Earth's geologic history, including climate reconstructions.

Marine deep biosphere research has greatly benefited from investigations by the scientific drilling community through the Integrated Ocean Drilling Program (IODP) and Ocean Drilling Program (ODP), and it has influenced the future scope of the international scientific drilling program (IODP, 2011). Historically, drilling programs concentrated on understanding the composition and diagenesis of deep-sea sediment, which allows for reconstruction of prior tectonic and oceanic current conditions, records indicators of past climatic variables, and allows for predictions of future shifts in currents and climates. Since the early 1990s, priorities of some drilling expeditions have expanded to include the collection of samples for microbiological study, allowing for preliminary analyses investigating aspects of the deep marine biosphere (Cragg et al., 1990, 1996, 1998; Whitman et al., 1998; Parkes et al., 2000; Kallmeyer et al., 2012). Initial observations indicate that microorganisms in this environment are capable of maintaining slow metabolic activity; however, details of specific rates in the marine deep biosphere are limited. This is partly due to limited sample collection and difficulties in making rate measurements; however, new advances are being made which will allow for a greater understanding of microbial activity in the marine deep biosphere.

## Recent advances in marine deep biosphere research

### Advances in sample collection and *in situ* experiments

Recent findings of spatial and temporal distribution and rates of microbial activity in the marine deep biosphere have been made possible by advances in sample collection and data analysis. Although scientific ocean drilling has continued since the late 1960s and routinely collects baseline geophysical and geochemical parameters from cored material, systematic collection of samples suitable for microbiological investigation lagged in development and application. One major development is the microbiological sampling protocols enacted within the last few years on IODP Expeditions that collect deep sediment and basement cores [example sampling protocols can be found in the *Methods* sections of recent expeditions' reports (Expedition327Scientists, 2011; Expedition329Scientists, 2011a)]. Adapting microbiological sampling and preservation strategies to the existing IODP workflow required establishing, for example, autoclaves and banks of ultralow temperature freezers on ships and in core repositories for preserving materials for sensitive DNA- and RNA-based methods. Both qualitative and quantitative contamination monitoring have developed in parallel with this routine collection (Smith et al., 2000; Lever et al., 2006), enabling greater confidence in the integrity of the recovered samples. Established protocols have also enabled a new era of sample collection for shore-based scientists via IODP's online sample request pipeline. Recent collections of deep biosphere samples are from a range of subsurface habitats including carbon-poor sediment (Expedition329Scientists, 2011b; Expedition336Scientists, 2012), young and old basalts (Expedition327Scientists, 2010; Expedition329Scientists, 2011b; Expedition330Scientists, 2011; Expedition336Scientists, 2012), gabbroic crust (Mason et al., 2010), carbon-rich sediment (Wehrmann et al., 2011), buried coral reefs (Expedition325Scientists, 2011), and hydrothermal systems above convergent plate boundaries (Expedition331Scientists, 2010). On-going studies with these samples will undoubtedly lead to new understanding of microbial activity and diversity in these disparate settings.

Other advances in sample collection have occurred outside of the conventional scientific ocean drilling programs that provide new opportunities for collecting deep biosphere samples. For example, revised giant piston coring devices for retrieval of longer (10 s of meters) sediment cores have been developed in both the USA (Røy et al., 2012) and France (Bourillet et al., 2007). Similarly, seabed rock drills recently developed by the UK (Petersen et al., 2007) and Germany (Freudenthal and Wefer, 2009; Krastel et al., 2011) enable the collection of zero-age crustal materials and hydrothermal vent deposits—something that is difficult to accomplish with conventional ocean drilling vessels which require soft substrate for establishing boreholes. In addition, new coring devices enable direct cultivation with deep sediment (Parkes et al., 2009a).

Development of a variety of short- and long-term seafloor and subsurface observatories specifically designed for microbiological research has opened a new window into the relatively understudied deep crustal environment (Edwards et al., 2012a). Installation of new Circulation Obviation Retrofit Kits (CORKs) that have been redesigned and constructed using inert materials such as Teflon-like fluid delivery lines, titanium fittings, and fiberglass borehole casing (e.g., Fisher et al., 2011a; Edwards et al., 2012b; Orcutt et al., 2012) have allowed for collection of high-integrity fluid samples at the seafloor during ROV expeditions (see Wheat et al. (2011) for recent review of CORK developments). Additionally, new seafloor instrumentation and subsurface observatory hardware has recently been deployed to enable microbiological investigations (Fisher et al., 2011a; Orcutt et al., 2011b; Edwards et al., 2012b), including an *in situ* electrochemical analyzer (e.g., Edwards et al., 2011a), *in situ* fluid pumping systems for collecting pristine fluids and particles from hydrothermal vents, plumes, and microbial mats (Breier et al., 2012), and new integrated sensor and sampling packages for ROVs during short-term sampling, and for automated instrument packages during year-long CORK deployments (Cowen et al., 2012). In the specific case of sampling basement formation fluids from CORK observatories, the capability for collecting large volumes of formation fluid (60 liters per dive) has enabled investigations for biogeochemical activities (Lin et al., 2012) and microbial diversity (Jungbluth et al., 2012). *In situ* enrichment experiments can also be deployed on and in CORKs to promote isolation and characterization of rock-hosted microbial communities (Orcutt et al., 2011b; Smith et al., 2011).

### Advances in biomass quantification

The last decade of deep biosphere research has witnessed major advances in the capability for quantifying microbial cell abundance. Initial efforts to quantify the number of microorganisms in the deep biosphere were based on cell densities determined manually by epifluorescence microscopy of carbon-rich sediment collected near land (Cragg et al., 1990; Parkes et al., 1994; Whitman et al., 1998). Based on a compilation of the data available, initial estimates projected that deep sediments contained 3.55 × 10^30^ total microbial cells, representing the majority of microbial cells on Earth and a little more than half the amount of carbon found in all of the plants on Earth (Whitman et al., 1998). These early studies on organic carbon-rich sediments (which presumably would have more cells than carbon-poor sediment) were already pushing the limit of detection of the epifluorescence microscopy methods that were used. Several advances in sample processing have now recently enabled cell enumeration in even lower biomass samples. These include developments in cell separation from the sediment matrix to concentrate biomass (Kallmeyer et al., 2008) as well as automation of cell counting (Morono et al., 2009). In addition to methodological improvements for cell enumeration, recent efforts by IODP and national funding organizations have enabled microbiological sampling at an increasing variety of sediment and oceanic regimes, including more offshore sites and sediments underlying low-productivity gyres. As recently reviewed (Hinrichs and Inagaki, 2012), biomass calculations are sensitive to the sample sets used, levered on carbon per cell and sediment volume (depth) assumptions. Re-visiting the earlier calculations (Whitman et al., 1998) and including cell counts from low biomass sites has resulted in a revised number of cells downward by an order of magnitude, from 3.55 × 10^30^ to 2.9 × 10^29^ cells globally in subseafloor sediment (Kallmeyer et al., 2012). Similarly, taking into account revised estimates of cell size, as cells in oligotrophic environments tend to be smaller than those found in carbon-rich settings, the amount of C estimated to be contained in these cells has changed from 303 to 4.1 Pg C.

There are still a large variety of issues and caveats involved in the current cell number and mass estimates for the deep biosphere. While recent developments and added study sites have provided for an improved understanding of microorganisms in the deep *sediment* biosphere, very little is known in terms of cell biomass in the underlying basaltic crust. Until we achieve some basic understanding of the distribution and numbers of Bacteria and Archaea resident in basaltic crust, the size of the deep biosphere will remain unknown. The few studies that have successfully sampled this challenging regime have shown that oceanic crust can be inhabited by an array of microorganisms (Santelli et al., 2008; Mason et al., 2010; Lever et al., 2013). How this will affect global estimates of microbial biomass remains unknown until a greater diversity of crustal regimes (different ages, alteration states, amounts of fluid flow, etc.) have been sampled for microbiology. Enumeration of cells in rock is not a trivial matter, and efforts are ongoing to develop the tools and techniques necessary for these measurements (Edwards et al., 2011b). Based on an energetic perspective, microbial primary productivity in basalt systems is estimated at 0.5 Pg carbon, or (very) roughly 2 × 10^24^ cells worth of carbon (Bach and Edwards, 2003). Based on assumed cell volume and rock pore space model calculations, others suggest a rock biomass of 200 Pg (Heberling et al., 2010). Any calculation of biomass standing stock, however, requires assumptions about growth efficiency, cell size, and biomass turnover times—as well as total volumes of habitable, altered basalt—which are very poorly constrained (see e.g., Santelli et al., 2008), and any one of which can alter total cell numbers by orders of magnitude. It is possible that crustal biomass could easily match or exceed the 4.1 Pg carbon estimated from sediment microbial biomass (Kallmeyer et al., 2012).

Another consideration regarding cells in the deep biosphere is the presence of bacterial spores. These can form a considerable fraction of biological material in the subsurface (Lomstein et al., 2012), but are often overlooked by conventional cell counts due to the inability of conventional DNA stains to penetrate the dipicolinic acid which composes a large fraction of spore material (Murrell, 1967). Whether spores are merely the seed for future generations, or whether they compose a large amount of global subsurface biomass, remains to be seen. The abundance and role of viruses in the deep biosphere are largely unconstrained, as well, although they have been analyzed in deep sediment (Middelboe et al., 2011) and correlated with bacterial density (Bird et al., 2001), in hydrothermal vent fluids (Anderson et al., 2013), and within cultured microorganisms from the environment (Engelhardt et al., 2011). Viral production and high viral particle to cell ratios have been noted in deep sediment up to 11 Ma (Engelhardt et al., 2013).

### Advances in DNA and RNA extraction, amplification, and sequencing

Efficient nucleic acid extraction from sediment and crustal material is imperative to culture-independent studies of the deep biosphere. Techniques for extracting DNA and RNA from subsurface samples have varied widely, from indirect approaches that require an initial cell separation from the matrix before cell lysis (Lloyd et al., 2013) to more direct *in situ* lysis methods that extract nucleic acids directly from the sample (Mills et al., 2012a). Each approach relies on physical (e.g., bead-beating, freeze-thaw), chemical (e.g., solvents), and enzymatic disruption of the cell membrane. A variety of commercially available kits have capitalized on these basic processes to aid in extracting either DNA or RNA, although recent advances in extraction methods have permitted efficient co-extraction of DNA and RNA (Mills et al., 2008, 2012a). Other variations in these methods may be needed to address differences in cell membrane structures in Archaea (Urakawa et al., 2010) and eukaryotes, including fungi (Orsi et al., 2013a), and viruses (Engelhardt et al., 2011). A challenge for the subsurface biosphere community is the selection of a common method of nucleic acid extraction and the means to compare results from different methods and samples.

In addition to advances in the bulk characterization of the microbial communities in deep biosphere samples, state-of-the-art single-cell-based and “-omic” techniques are poised to yield important new insights into the functioning of microorganisms in these habitats. For example, single cell genomics (Stepanauskas and Sieracki, 2007; Stepanauskas, 2012) has already enabled functional determination of a ubiquitous and abundant group of presumably heterotrophic sediment Archaea (Lloyd et al., 2013). Single cell-based techniques are expected to be increasingly used to understand the functional potential of the vast phylogenetically-novel pool of deep subsurface microorganisms, including eukaryotes. Comparative analysis of genomic data, obtained from single cells or whole communities, can give indications of the specific adaptations that subsurface microorganisms utilize to exist in energy-challenged locations. One recent study illustrated this by linking functional genes and metabolic function in subsurface sediment communities (Lever et al., 2013). Another recent study of rRNA from eukaryotes showed that active fractions of fungi can be distinguished from likely inactive eukaryotes through comparative analysis (Orsi et al., 2013a). Pushing the limit of detection boundary even farther, recent work has also demonstrated the application of transcriptomics in deep biosphere samples for learning about microbial activity and growth in the subsurface (Orsi et al., 2013b).

### Advances in activity measurements

Some microbial activity measurements can be directly quantified using stable- and radio-isotope tracer-based techniques. For example, to measure the rate at which sulfate-reducing bacteria consume sulfate in sediment, trace amounts of ^35^S-labeled sulfate is added to the sediment, which is then incubated for a defined period of time [at *in situ* temperatures and pressures when possible; (Jørgensen 1982; Kallmeyer and Boetius, 2004; Jørgensen et al., 2006)], and then the amount of ^35^S-labeled sulfide produced from sulfate reduction is quantified to generate a turnover rate. Similar activity measurements have been conducted to study methane production (i.e., tracking the conversion of ^14^C-labeled bicarbonate or acetate into ^14^C-labeled methane) and methane oxidation [using ^14^C-labeled methane; (Joye et al., 2004; Orcutt et al., 2005, 2010)]. More recently, methods have also been developed to measure hydrogenase activity in marine sediments using tritiated hydrogen (Nunoura et al., 2009; Soffientino et al., 2009). Rates of activity are often expressed as moles of substrate consumed per volume (or weight) of material per time, which can then be scaled to an areal rate of activity (moles consumed per area of sediment per time). Potential limitations on activity measurements conducted in this fashion include impacts on microorganisms stemming from temperature and pressure changes (both during sample collection and incubation), and very low levels of activity that fall below the limit of detection of even the most sensitive isotopic techniques. Notably, tracer-based measurements of activity are quite rare in deep biosphere environments. For example, the Figure 1 map of locations where radiotracer measurements have been made underscores their limited global distribution. As will be discussed below, the development of either *in situ* rate measurement techniques, lowered limits of detection, or ways to standardize rate measurements are necessary to enable broader measurements of levels of microbial activity in deep sediment.

The activity of the deep biosphere can also be quantified based on the modeling of vertical profiles of chemical reactants and products of known microbial reactions. Pore water chemical profiles reflect the combined result of diffusive and/or advective transport of those compounds (advection is typically negligible in deep sediment), as well as the sum of production and consumption reactions (whether abiotic or biotic). For example, the activity of sulfate reducers is approximated from measuring the profiles of sulfate and sulfide in sediment and then using these data in reactive-transport-diagenetic models (Berner, 1980). Similar work has been done to model methane cycling in deep sediments (Claypool et al., 2006; Sivan et al., 2007). User-friendly analytical models to convert chemical gradients into activity determinations are becoming increasingly available (Berg et al., 1996; Aguilera et al., 2005; Thullner et al., 2007; LaRowe et al., 2008; Regnier et al., 2011).

In addition to the advances in sample collection highlighted previously, advances in *in situ* chemical sensors have begun to shed light on the activity of the subsurface biosphere. New *in situ* mass spectrometers, laser absorption spectrometers and electrochemical platforms now allow detailed chemical concentrations (and stable isotope composition) to be measured under *in situ* conditions (Glazer and Rouxel, 2009; Wankel et al., 2010, 2011, 2013; Edwards et al., 2011a; Cowen et al., 2012). For example, simultaneous measurements of multiple dissolved gases (via *in situ* mass spectrometry), combined with fluid flow rate measurements, revealed deficits of dissolved hydrogen, which were used to estimates rates of oxidation by the subsurface biosphere (Wankel et al., 2011). With a current shift toward autonomous, remote and cabled observational platforms, these types of technological advances will enable a core focus of deep biosphere research.

**Figure 3:**
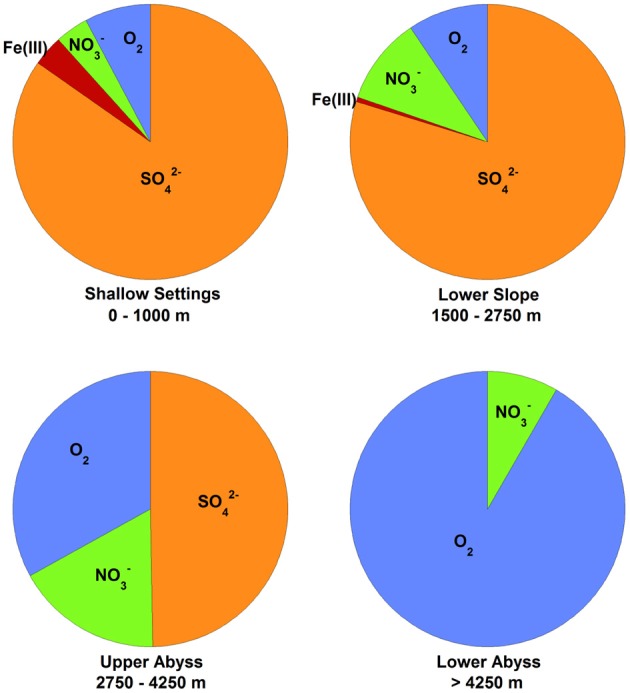
**Global marine sediment organic matter oxidation by electron acceptor and habitat, based on data published elsewhere (Thullner et al., 2009) and reprinted here with permission**. Pie chart sections represent the percentage of organic matter delivered to the seafloor that is oxidized by the indicated electron acceptor in the upper 50 cm of sediment. The depths of the seawater-sediment interface for each environment are listed below each chart. Sufficient data were not reported to include Mn(IV) as an electron acceptor, but note that it is generally only 10% of the values for Fe(III) (Thullner et al., 2009). Other fates of organic matter degradation (e.g., fermentation and methanogenesis) were not considered in the study [methanogenesis accounts for about 5% of global carbon mineralization (Jørgensen and Kasten, 2006)].

**Figure 4:**
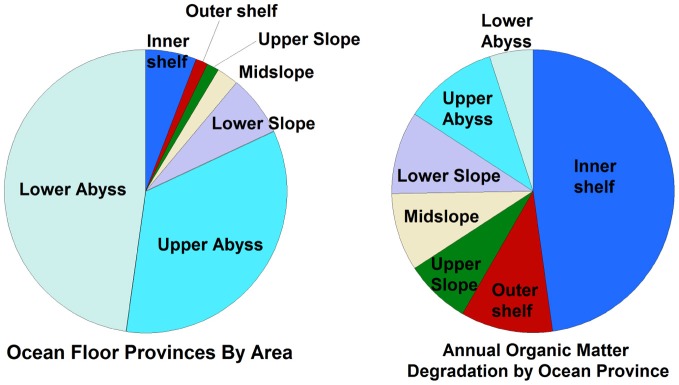
**Relative percentage of different sedimentary habitats (by area) compared to the relative amounts of cumulative organic matter degradation in those habitats, based on data presented elsewhere (Thullner et al., 2009) and reprinted here with permission**. Note that only habitats between 60°N and 60°S were considered in this study, and that the study assumed that all organic matter was degraded in the upper 30 cm of sediment.

Several important findings have emerged from the limited analysis of microbial activity in the subsurface using both tracer- and model-based methods. As summarized in Figure 2, the range of microbial activities (expressed in mol e- transformed per year per liter of material) in the subsurface spans more than thirteen orders of magnitude, with much higher volumetric rates in energy-rich environments, such as hydrothermal vent ecosystems with abundant hydrogen (Wankel et al., 2011), and very low rates in energy-poor environments, such as extremely oligotrophic sediment (Røy et al., 2012). Although it is often assumed that the lowest volumetric rates of metabolic activity are from deep and extremely oligotrophic sediment in the southern Pacific (Røy et al., 2012), this is not necessarily true on a per-metabolism basis (Figure 2). Global trends in activity in marine sediment based on energy-availability is discussed in greater detail elsewhere (D'Hondt et al., 2009). Figure 3 provides a summary of the dominant electron acceptors used by sediment microorganisms to fuel organic matter breakdown according to habitat type, based on data presented elsewhere (Thullner et al., 2009). Notably, organic matter oxidation in near-shore sediment is predominantly fueled by sulfate reduction, whereas oxygen and nitrate become the dominant terminal electron acceptor with distance from land and increasing water depths (and thus, distance from land-based organic matter and nutrient inputs to fuel primary production). Considering the global distribution of these various habitat types (Figure 4), it is remarkable that the majority of organic matter degradation occurs in the shallow, near-land environments, even though these environments comprise a relatively small fraction of the areal distribution. Conspicuously absent from all of these surveys are data from polar regions, however (Figures 1, 4).

### Advances in understanding energy supply and demand in the deep biosphere

Although it is becoming clear that microorganisms are abundant in deep marine settings (Parkes et al., 1994; Whitman et al., 1998; Cowen et al., 2003; D'Hondt et al., 2004; Edwards et al., 2005; Schippers et al., 2005; Santelli et al., 2008; Kallmeyer et al., 2012), it is unclear how active they are (Jørgensen, 2011). Determination of this activity is made difficult by the size and diversity of subsurface habitats, their relative inaccessibility and the difficulty of cultivating representative microbes. However, theoretical and modeling techniques have been used to investigate some of the variables that affect activity in these systems. In particular, thermodynamic models have been used to quantify the energy supply and demand in various ecosystems since energy availability is one of the key factors that affects microbial activity levels (Van Briesen, 2002). Furthermore, quantification of deep biosphere energy budgets can be used to infer what types of reactions microorganisms are catalyzing and the amount of biomass that can be sustained under a given set of environmental conditions.

Active microorganisms require energy that is ultimately harvested from the catalysis of redox reactions. The amount of energy available from these reactions with a given set of environmental conditions can be determined by calculating the Gibbs energies of potential catabolic reactions. This procedure can determine what geochemical variables (e.g., temperature, pressure, pH, salinity, composition) control the system, quantify the energetic potential in the deep biosphere, and help identify the likely electron donors and acceptors that are being used by a microbial community. These conditions are influenced by reaction rates and the diffusive and advective transport of reactant and product species, which are in turn governed by the porosity, permeability and mineralogy of a given locality. As a result, in order to understand the dynamics of the deep biosphere, the geological and geophysical parameters that describe it must also be taken into account. Energetic profiling has been successfully carried out in submarine hydrothermal settings (Shock et al., 1995; McCollom and Shock, 1997; McCollom, 2000; Amend and Shock, 2001; Shock and Holland, 2004; McCollom, 2007; LaRowe et al., 2008) shallow marine and terrestrial hydrothermal systems (Amend et al., 2003; Inskeep and McDermott, 2005; Inskeep et al., 2005; Rogers and Amend, 2005; Spear et al., 2005; Rogers and Amend, 2006; Rogers et al., 2007; Skoog et al., 2007; Windman et al., 2007; Costa et al., 2009; Shock et al., 2010; Vick et al., 2010) and, to a lesser extent, in ocean sediment (Schrum et al., 2009; Wang et al., 2010) and basement rock (Bach and Edwards, 2003; Cowen, 2004; Edwards et al., 2005; Boettger et al., 2012). However, a comprehensive assessment of the amount and type of energy sources in deep marine settings has yet to be carried out.

The amount of energy required by a microbial community is largely a function of the metabolic state of the ecosystem. That is, in the presence of a sufficiently large amount of energy, microorganisms synthesize organic compounds for growth and for extracellular functions such as communication, nutrient acquisition, physical support and stability. However, under low-energy conditions, active microbial synthesis is limited to maintaining cellular integrity through biomolecular repair and replacement, a collection of activities referred to as maintenance (Tijhuis et al., 1993). When energy and/or nutrients are essentially not available, many microorganisms enter into a dormant or survival state (Price and Sowers, 2004). Determining the energetic regime of a given microbial community is largely a function of both how much energy is available (Van Briesen, 2002) and how much energy is required to synthesize biomass under the conditions specified by the geochemical environment. Although it is difficult to ascertain which of these states describes the microorganisms in a particular deep biosphere setting, the amount of energy required to synthesize a broad range of biomolecules under biologically relevant conditions can be quantified. In particular, the thermodynamic data required to do this is now available for amino acids, fatty acids, carbohydrates, nucleotides, coenzymes and unfolded polypeptides (Shock, 1995; Helgeson et al., 1998; Richard and Helgeson, 1998; Dick et al., 2006; LaRowe and Helgeson, 2006a,b; Amend and Plyasunov, 2001; McCollom and Amend, 2005; LaRowe and Dick, 2012).

The amount of energy that is available from catabolic reactions determines how fast microorganisms grow, and thus the rate and quantity of biomass produced in a given setting. In a low-energy environment, which describes most deep biosphere habitats, the relationship between energy supply, energy demand, and the rates of microbially catalyzed processes are unclear (Jørgensen, 2011). Additionally, although energy might be available in a particular setting, it might not be enough to stimulate microbial activity (Jin and Bethke, 2002, 2003, 2005, 2007, 2009; Hoehler, 2004; Bethke et al., 2011). It is unclear at what energy level microorganisms living in low-energy subsurface settings switch among dormancy, maintenance activities, and growth.

## Future research directions

Obtaining samples and maintaining them at *in situ* conditions is difficult for deep biosphere work and requires advancement of technologies to either make measurements *in situ* or to maintain a sample at *in situ* conditions. Technologies of the latter have been used for some time, including the pressure core sampler used in drilling programs (Parkes et al., 2009a,b), and the biomass recycle reactor that stimulates subsurface starvation conditions (Colwell et al., 2008) although widespread usage is lacking. Yet for the former, technologies are just being developed. For example, studies showing successful deployments of underwater mass spectrometry reveal *in situ* concentrations of different constituents (Camilli and Duryea, 2009; Wankel et al., 2011). Taking these measurements to the next level to measure stable carbon isotope ratios can help decipher active processes occurring in sediment (Wankel et al., 2011). Designing these systems to be able to sample pore-fluids within sediment will require continued collaborations between scientists and engineers.

### Need for standardization and routine measurements

The volume of biological data being collected from the subsurface has rapidly increased over the past decade due, in large part, to greater emphasis on exploring the biosphere by groups like IODP and the Center for Dark Energy Biosphere Investigations (C-DEBI). More laboratories are requesting samples and are using new, diverse culturing and molecular techniques, but each laboratory is attempting to answer similar questions about the diversity, extent, and function of life in the subsurface. These questions are too large for a single laboratory; therefore, a collective effort is required. However, changes in sampling techniques and experimentation protocols can inadvertently bias results and reduce the overall cross comparison potential between studies (Mills et al., 2012a). Before the subsurface biosphere community becomes too large, we have a chance to develop a degree of standardization that will allow for true, meaningfully global comparisons to occur.

While the international research community has many options for obtaining subsurface samples, one of our assets is the ability to capitalize on resources within IODP. Geochemical and geophysical research objectives represented on IODP expeditions are routinely provided by dedicated shipboard scientists and technicians assigned to completing standard procedures on all core material. The call for an additional biological workload on these individuals is typically met with an argument claiming a lack of time and resources onboard. The need for standard and routine biological procedures must be viewed not as an added burden on the shipboard party, but as a necessity to complete expedition and post-expedition objectives. If equipment and personnel do not currently exist with the capacity to complete standard biological analyses, IODP should take this as a charge to better equip ships with a fully functional laboratory and science party. The main areas where standardization of biological shipboard procedures would make an immediate impact are with core material sampling and cell counting.

A change in standard coring protocol should be adopted to provide samples suitable for biological analysis. Standard use of a contamination detection protocol will provide a higher level of confidence for all downstream analysis. These contamination tests should also be of high interest to chemists, as their results can be compromised as well during the drilling processes. This protocol should be initiated during the drilling process to provide the greatest potential to determine drilling-related contamination of the core material. Advanced testing of current contamination tracers and proposed novel ideas can be conducted on any expedition provided the right personnel are onboard and available for analysis. Core material will then be available both to the onboard science party and for projects during post-expedition research. Many of the cores currently being stored are not available for biological analysis because such procedures were not in place. While this request for better monitoring appears to be straightforward, time and financial costs associated with such procedures have limited the use of current technologies. However, we emphasize that the scientific gain and necessity to facilitate biological and chemical studies should be considered in these cost analyses.

Substantial advances have been made in cell enumeration over the past several years so that a routine method may now be adopted. The method described by Morono et al. (Morono et al., 2009) is both sensitive to low cell counts and reproducible across a wide range of sediment types. One of the key components to this method is a cell extraction step used to separate cells from sediment particles. In methods that do not remove the sediment, detection limits are 10^4^ to 10^5^ cells ml^−1^ while the new method promotes a 10^3^ cells ml^−1^ detection limit. A recent concern with this method has been the capacity to count spores, viral particles and micro-eukaryotes, including fungi that may have different sizes and densities compared to the typically targeted prokaryotic cells. This concern reflects a growing trend to expand what is characterized and included in the subsurface biosphere. Refinements to this method may need to be considered, however, these issues do not outweigh the benefit from a standard shipboard cell count being performed. Time commitments and variability between technicians and expeditions has been reduced by the procedure being automated. Through a concerted effort to standardize the cell counting method, we will be able to better compare samples from future expeditions and begin to ask global questions about cell abundance.

A step in the overall goal of method standardization needs to be a discussion resulting in a decision to determine a basic diagnostic gene target for characterizing microbial community structure. While there is little questioning the value of the small subunit (SSU) rRNA gene for prokaryotic taxonomic description, the choice of region within this conserved gene has been widely disputed, with the main arguments being between the V1–V3 and the V4–V6 hypervariable regions. Both of these regions have biases and benefits (Youssef et al., 2009; Kumar et al., 2011) and both have well-developed primer sets used for amplification and quantification. As more groups begin to use high-throughput, next generation sequencing, the community has the opportunity to determine the best region to target so that future studies can be better compared. Biogeographical descriptions of the subsurface community are most effective when variables between analysis procedures are reduced. Choosing a standard region for comparison will not restrict research groups from analyzing additional, more taxonomically specific regions. However, by collectively targeting a single sequence, we will begin to assemble a more robust dataset for examining global trends in microbial diversity and begin to address the question of overall diversity within the subsurface. Having these conversations while the community is well-connected and focused on building the field will benefit subsurface research as a whole.

Standard methods for determining and reporting rates of *in situ* processes such as sulfate reduction, methanogenesis, and anaerobic methane oxidation have been used for many decades, although the limit of detection for these methods is an important consideration (Kallmeyer et al., 2004), as is the logistically challenging use of radioisotopes needed for these analyses. The deep biosphere community as a whole can adopt these methods to help standardize analysis for better direct comparison to occur. At the very least, adopting a consistent unit that is commonly reported would be helpful. For example, measured or inferred rates of microbially catalyzed reactions should be reported in units that are comparable and easy to translate from one metabolic pathway to another. For instance, by using units of mol e- transferred cm^−3^ yr^−1^, catabolic activities such as aerobic heterotrophy can be directly compared to chemolithotrophic reactions. Furthermore, if rate measurements are carried out under pressure, temperature or composition conditions different from those characterizing the sample location, then this should be noted and referred to as *apparent* rates of catabolic activity. While few rates of microbial activity have been measured globally (Figure 1), the incorporation of deep biosphere objectives into the IODP goals has helped advance the ability to make these measurements and stressed the need to continue to measure rates on future expeditions.

### Analog systems and manipulation experiments

The use of *in situ* manipulation experiments is now common in terrestrial subsurface microbiological research, where this class of studies can be used to examine the outcome of intentional or inadvertent changes to subsurface environments. Where bioremediation is seen as a solution to subsurface contamination, so-called “push-pull” studies have been used to interrogate the *in situ* activities of microorganisms called upon to metabolically degrade organic chemicals (Istok et al., 1997; Schroth et al., 1998) or immobilize inorganic chemicals (Colwell et al., 2005; Fujita et al., 2008). In low temperature environments, such as shallow permafrost settings that are susceptible to warming trends, recent work has examined how microbial community activities respond to artificially induced thermal pulses (Mackelprang et al., 2011). Microbial transport (Harvey, 1997) or “mark-and-recapture” studies in continental settings provide inspiration for what could be done below the seafloor. For example, native subsurface cells were cultivated from sediment and then grown in the laboratory in the presence of a ^13^C labeled substrate. These ^13^C-heavy cells were then released into their native setting in the presence of other indigenous microorganisms and then tracked as they migrated by measurement of the ^13^C signal using mass spectrometric analysis (Holben and Ostrom, 2000). Growth or activity during the underground transport and metabolism by the re-introduced cells could be inferred by loss of the ^13^C-label in recovered cells.

Despite the inherent complexity of moving from a terrestrial to a deep marine setting, we envision that similar technologies may be applied to subseafloor environments that are susceptible to changing conditions or where fluid movement is common in the subsurface. Examples of the former condition are locations where active tectonics or state changes in the physical sedimentary features (e.g., formation or decomposition of hydrates) in the sediment may induce dynamic changes. In the latter case, fractured rocks or crustal materials in the seabed offer a chance to evaluate how microbes that exist within a regime of fluid movement respond to low levels of reductants. Sophisticated versions of CORKs might allow direct injection or withdrawal of fluids with prescribed chemistries in the seafloor in order to test hypotheses related to how the subsurface microbial communities will respond. Steps in this direction have occurred with the recent experiments performed on the Juan de Fuca flanks CORK network (Fisher et al., 2011b), building on the legacy of research at this location documenting the dynamic nature of microbial communities in ridge flank crust after being disturbed by drilling (Cowen et al., 2003; Nakagawa et al., 2006; Orcutt et al., 2011b; Jungbluth et al., 2012; Lin et al., 2012). A next step could conceivably be the introduction of reactive tracers to determine how the microbial communities along the flow-path alter the introduced compounds and the rates at which these alterations occur. Another possibility would be the co-location of a subseafloor manipulation experiment and one of the regional scale nodes of the new Ocean Observatories Initiative (OOI). This would open possibilities for linking subsurface processes with the surface where there is the possibility of exchange between the two realms.

The idea of observatory-enabled manipulation experiments builds off of the history of twenty-eight instrumented borehole observatories (i.e., CORKs) that have been deployed during the past two decades (Wheat et al., 2011). This burgeoning global network of CORKs provides a range of hydrologic and geologic settings from hydrothermal systems to hydrate complexes and from active spreading centers through ridge flanks to subduction zones. These settings represent a range of environmental conditions—e.g., from acidic (pH 5.1) to basic (pH 12.5), from hot (265°C) to cool (3°C), from serpentinite- to basalt- to sediment-hosted (Wheat et al., 2011)—that shape microbial community structure and activity. Even with this seemingly large number of CORKs and the range of conditions that they represent, half of these CORKs are within a 100 km cluster of the six CORKs that overlie ~3.5 M basaltic crust on the eastern flank of the Juan de Fuca Ridge. Such a small footprint barely begins to provide a representation of global crustal conditions. Recent developments in sealing and instrumenting “legacy” boreholes (Wheat et al., 2012) open up the possibility of re-visiting dozens of potentially suitable boreholes started in earlier drilling expeditions and upgrading them to observatories, as has been proposed elsewhere (Edwards et al., 2012c). This capacity could allow many more crustal, biogeochemical and physical conditions to be monitored or manipulated for understanding the spectrum of subsurface diversity and activity. An important consideration for the scientific community will be cost-benefit analysis of long-term observatories, which can cost from hundreds of thousands to millions of dollars to install coupled with the lifetime costs of return visits for sampling and servicing (Wheat et al., 2012). Connection of long-term observatories to deep sea cabled networks, as is being done with the NEPTUNE Canada program and the Endurance Array of the Ocean Observatory Initiative, is another important consideration.

## Summary and outlook

The past decade has seen substantial changes in our understanding of the size, diversity, novelty, and importance of the deep marine biosphere, and the next decade of deep biosphere research within the International Ocean Discovery Program (IODP, 2011) and other national programs is poised to yield new insights and fundamental discoveries. Determining how slowly growing microorganisms in the deep biosphere survive for extremely long periods of time is a major research frontier, as is determining the size and dynamics of microbial ecosystems in upper oceanic lithosphere. Research in both of these directions, and others, will also open up novel habitats and vast microbial diversity to exploration for natural product research, an untapped resource in the deep subsurface.

### Conflict of interest statement

The authors declare that the research was conducted in the absence of any commercial or financial relationships that could be construed as a potential conflict of interest.
